# Emotional Intelligence and Delivering Bad News in Professional Nursing Practice

**DOI:** 10.7759/cureus.40353

**Published:** 2023-06-13

**Authors:** Daniel A Nnate, Abdulqadir J Nashwan

**Affiliations:** 1 Urgent Care Division, Countess of Chester NHS Foundation Trust, Chester, GBR; 2 Nursing Department, Hamad Medical Corporation, Doha, QAT

**Keywords:** staff wellbeing, nurse retention, nursing practice, emotional intelligence, burnout, bad news

## Abstract

Delivering bad news often impacts nurses’ emotional well-being and relationships with patients. However, most practice models do not offer a sufficient solution to the distress and reduced job satisfaction that may thus arise. This paper offers a critique of theoretical frameworks for breaking bad news in clinical settings, with the aim of highlighting the inadequate evidence available to guide nursing practice with regard to managing the emotional burden of breaking bad news. Firstly, the concept of emotional intelligence is introduced, followed by an overview of the impact of delivering bad news on the psychological well-being of healthcare workers. Several models for delivering bad news in clinical practice were then presented to emphasise the lack of evidence regarding ways of mitigating the burden associated with breaking bad news. Key components of emotional intelligence are highlighted to increase awareness of this factor among nurses and enable them to improve their interpersonal skills to mitigate the impact of breaking bad news. Enabling nurses to develop emotional self-awareness before utilising these frameworks will likely lead to increased nurse retention rates and improve reflective practice and communication skills, which could, in turn, strengthen nurse-patient relationships and subsequent care planning.

## Introduction and background

The concept of emotional intelligence has been applied across several disciplines and received support in healthcare [1,2]. Emotional intelligence entails managing one's own emotions to relieve stress, communicate effectively, and overcome barriers, which are predictive factors concerning the behaviours and attitudes exhibited by nurses when responding to the holistic needs of patients [1,2]. Over the years, the nursing profession has witnessed a shift from enrolling candidates with high academic grades to individuals with good virtues, values, attitudes, and competencies in rational decision-making [3]. While these characteristics are required for the delivery of person-centred care, Rankin [2] further advocates that, rather than selecting candidates based on their values, more emphasis should be placed on the task of enabling nurses to recognise the impact of their values on their emotions. Therefore, awareness of one's emotions may be regarded as the key to holistic health care delivery. Such an approach may be a response to the need to manage the emotional distress entailed by the task of caring for patients. Hence, more emphasis has been placed on emotional intelligence in nursing practice.

According to Buckman [4], the term "bad news" in healthcare refers to any information that encourages a patient to have a negative view of the future. The definition of bad news developed in this seminal literature continues to apply today, and breaking bad news to patients has also become an essential nursing skill that often constitutes part of the standards of proficiency for registered nurses [5,6]. Research has shown that breaking bad news has a significant negative emotional impact on healthcare professionals because of the attachments they develop with patients and families over time [5]. Consequently, a significant patient interaction concerning a diagnosis of death based on the patient’s disease prognosis often leads to psychological distress and decreases compliance with further anticipatory care. That fact notwithstanding, the psychological impact of having to deliver such news may lead to emotional distress among healthcare professionals.

Most of the literature associated with distressing patient-caregiver communication in practice is in response to the need to mitigate the high risk of burnout and compassion fatigue associated with caring for terminally ill patients [5,6,7]. Bousquet et al. [5] also reviewed the literature on the task of delivering bad news in oncology and found that experience and emotional stability are associated with the level of perceived burnout as well as distressing effects on performance and general health. These authors further concluded that the ability of caregivers to adapt to each patient’s feelings and health situation depends solely on the empathic abilities of the health professionals involved. This current study aimed to explore current clinical guidelines for delivering bad news in professional nursing practice. Subsequently, theoretical frameworks for breaking bad news were evaluated to highlight the gaps in the current protocol regarding the lack of evidence about ways of mitigating the emotional impact of breaking bad news.

## Review

Strategies for breaking bad news

Over the years, several models for the delivery of bad news in clinical practice have been developed. The most common models that have been widely represented in the literature are as follows: SPIKES (setting, perception, invitation or information, knowledge, empathy, and summarise or strategize) [7]; PEWTER (prepare, evaluate, warning, telling, emotional response, regrouping preparation) [8]; BREAKS (background, rapport, explore, announce, kindling, and summarise) [9]; ABCDE (advance preparation, building a therapeutic relationship, communicating well, dealing with patient and family reactions, and encouraging/validating emotions) [10]; and Kayes’ 10 steps (1. preparation; 2. what does the patient know?; 3. is more information wanted?; 4. give a warning shot; 5. allow denial; 6. explain; 7. listen to concerns; 8. encourage the ventilation of feelings; 9. summary-and-plan; 10. offer availability) [11] (Table 1).

**Table 1:** Models for communicating bad news

	Plan: Preparatory phase	Communicate: Delivering the bad news	Response: Building a therapeutic relationship	Follow-up: Offering emotional support and care-planning
SPIKES	Setting	Perception, invitation, information, knowledge	Empathy	Summarise or strategize
PEWTER	Prepare, Evaluate	Warning, telling	Emotional response	Regrouping preparation
BREAKS	Background	Rapport, announce, explore	Kindling	Summarise
ABCDE	Advance preparation	Building a therapeutic relationship, communicating well	Dealing with the patient and family reactions	Encouraging/validating emotions
Kayes’ ten step	1. Preparation	2. What is known?; 3. Is more information wanted?; 4. Allow denial; 5. Give a warning shot	6. Explain (if required); 7. Elicit concerns; 8. Venting of feelings	9. Summary and plan; 10. Offer availability

The SPIKES protocol is the most commonly used model and was initially developed for breaking bad news to cancer patients [7]. This process has been adopted for use by clinicians in different clinical areas, which led to the development of modified versions [12]. The PEWTER model, originally developed for use by school counsellors, has been adopted by emergency physicians for breaking bad news [8,13]. Güneri et al. [14] identified the ABCDE framework as an alternative to the SPIKES model; each letter represents a phase in the six-step sequence. The authors also suggested that the six-step ABCDE protocol for delivering bad news can be applied in various healthcare professions, including dentistry [14]. The BREAKS and Kaye's protocol have also been highlighted in recent literature [12]. Kaye's 10-step approach further emphasised the need to clarify information for patients and deal with emotions as they arise. Table 1 highlights the similarities in the designs of several models for breaking bad news.

The aforementioned models for breaking bad news in clinical practice tend to follow a similar path and can be easily adopted by clinicians. Although these procedures employ a similar framework involving planning, communication, response, and follow-up, they also require the healthcare professional to prepare for such interactions and anticipate any response that the patient is likely to exhibit. Furthermore, offering emotional support thereafter with a potentially hopeful and positive outlook for the patient is also emphasised in these models [15,8]. Considering the breakdown of the stages involved in the delivery of bad news, it may be conceivable to suggest that there is no evidence that these protocols currently support the emotional well-being of nurses. Bumb et al. [15] instead highlighted the fact that adopting an evidence-based protocol for breaking bad news may inspire confidence in nurses. Nevertheless, the knowledge gap that exists because of the insufficient literature on the emotional burden associated with breaking bad news suggests that there may be inadequate evidence to guide nursing practice concerning the concise steps that should be followed. Hence, these strategies for breaking bad news may not be suited for nurses who strive to meet the patient's care needs holistically.

Critique of theoretical frameworks for breaking bad news and incorporating values into guidelines

The PEWTER model acknowledges the emotional distress experienced by both the practitioner and the patient [8]. The importance of maintaining a relaxed mood to successfully convey the news and proceed to the subsequent phase was also stressed by these authors. Although this model recognises the emotional impact of delivering distressing news and the need to provide strategies that can be used to cope with perceived stress, there is little evidence of the use of this approach in clinical practice. On the other hand, the SPIKES model, which was initially designed to reduce burnout among healthcare professionals, has frequently been referenced in clinical guidelines [16]. It is, however, stressed that addressing this challenge was not incorporated into the six-step approach included in the protocol [16]. While the SPIKES protocol is often perceived as the preferred model for breaking bad news, there is no robust evidence to support its ability to prepare nurses for the emotional burden associated with breaking bad news and the resulting satisfaction. The application of this approach in nursing practice has also been the subject of professional criticism [16,17].

Warnock et al. [17] further advocated that the SPIKES framework, designed for medical consultation, may be less suited for nurses. The reason is that this approach does not consider the emotional consequences that this task may have on the nurse both before and after the 'news' has been delivered. Because of the nature of the nurse-patient relationship, nurses may not be prepared to discuss bad news regarding the patient's diagnosis or disease prognosis frequently. Additionally, nurses are always in contact with the patients, and due to the presence of such good rapport, patients can ask questions about their health conditions at any time. Such a conversation is likely to arise when the nurse supports the patient in the activities of daily living, such as when administering medication or performing other activities aimed at keeping the patient comfortable. Hence, even though the SPIKES approach is recommended to nurses as a model of good practice, it may not always be applicable in clinical settings.

The evolution of a value-based nursing workforce advocates that nurses should focus on patients' emotional and physical well-being, following professional and organisational values [2]. Guided by their morals, nurses are perhaps considered advocates for the patient's rights and are expected to work efficiently to deliver excellent care. While nurses are often confronted with the need to consider "positive patient experience" and the "personal reward gained from job satisfaction" in their decision-making, the desired positive outcome following the delivery of bad news may only be achieved when the nurse's emotional state is taken into consideration. To promote concordance with further rehabilitative and anticipatory care after the disclosure of sensitive information, ethical issues often emanate from decisions to either uphold the patient’s right to information regarding their health condition or to avoid encounters that could lead to such conversations. However, regarding the delivery of holistic care, the latter is never an option for nurses.

Disclosing a life-changing diagnosis, prognosis, or complicated medical procedure to a patient is often a challenge for healthcare professionals. Bullock [18] asserted that health practitioners often struggle to disclose information to patients regarding their diagnosis, especially in terminal cases. These findings are similar to those reported by Bousquet et al. [5]. In this context, the authors also stressed the need to balance the amount of information that is concealed in an attempt to promote optimism regarding the patient’s future. In addition, the Patient Right to Know Act [19] places a great deal of emphasis on the patient's autonomy regarding information relevant to their well-being. However, disclosing bad news to a patient regarding their health often leads to a dilemma between providing accurate information and the moral obligation to maintain the patient's hopeful attitude towards their health.

Bullock [18] further argued that an informed patient is in a better position to make good decisions regarding their health and that withholding information from patients for paternalistic reasons may not conform with the principles of beneficence. Thus, to ensure patients’ rights, the International Council of Nurses [20] code of ethics demands that patients’ autonomy be preserved by providing the patient with sufficient information when needed. Because the patient-nurse relationship involves honesty, trust, and empathy, it is necessary to protect nurses from the emotional breakdown associated with the delivery of bad news. Knowledge of how emotions affect our judgements, behaviours, and actions is considered to be key to understanding how nurses connect with patients in response to their health needs.

The proposition of a modified framework for delivering bad news

Emotional intelligence includes five components: self-awareness, self-regulation, motivation, empathy, and relationship management [1, 21]. Self-awareness allows an individual to obtain a clear picture of their strengths and weaknesses. This attribute, which is an element of reflective practice in health care, can be developed through reflection-in-action (which is performed on the spot and is often known as "thinking on one’s feet) and reflection-on-practice (thinking about an experience in retrospect). Self-awareness offers the opportunity for self-regulation, which helps the individual keep their emotions in check and further prevents nurses from making irrational decisions. While motivation promotes enthusiasm for continuous personal development, by constantly striving to improve interpersonal skills, nurses are encouraged to work on their emotional awareness. Empathy enables the nurse to be in tune with the patient’s emotions. Good listening skills are key to the establishment of an empathic relationship, as such skills instil trust in the patient-nurse relationship. Kooker et al. [1] emphasised the fact that active listening and taking an interest in the patient’s concerns further enable nurses to manage patients’ health needs more effectively.

Delivering bad news entails certain emotional commitments; however, not many actions have been taken to minimise the effect of this process on nurses. In the process of establishing empathy with a patient, nurses may find it difficult to detach themselves from the patient’s experience. Thus, proficiency in emotional intelligence may play an important role in supporting nurses when delivering bad news during their practice learning. Because reflection-in-practice often occurs spontaneously when the nurse is involved with the situation at hand, developing this skill may require prior experience. In response, several authors have highlighted the need to develop protocols that consider the patient’s emotional and health outcomes and those of health care professionals [5, 16]. Therefore, enabling nurses to develop emotional self-awareness before utilising protocols for breaking bad news is likely to improve their communication skills and reflective practice, reducing their emotional burnout.

Kooker et al. [1] asserted that competencies in each of the components of emotional intelligence can be learned just like any other personal skill. Enhancing one’s competencies in emotional intelligence through theoretical and simulation-based learning has also been recommended for combating the empathy drift, exhaustion, and feelings of ineffectiveness experienced by healthcare personnel [22-24]. Considering the need to incorporate emotional intelligence into frameworks for breaking bad news, a major area of concern lies in the planning for and response to this process. These stages are emotionally daunting for healthcare professionals [15, 16]. Overall, the preparatory phase entails reviewing the patient’s medical history, determining whether the patient will require emotional support from friends or relatives, and identifying a suitable time and environment for delivering the news. While delivering bad news involves communication, the response and follow-up phases require the establishment of a therapeutic relationship, the provision of emotional support, and planning for further care (Table 1). It is therefore suggested that the nurses’ ability to reflect on the impact of delivering such bad news on their well-being and their relationships with others before engaging with the patient can prepare nurses to devise means of mitigating any unforeseen emotional outbursts that may arise during this process.

## Conclusions

This paper has argued that an awareness of the emotional consequences of bad news paves the way for communication to occur in a systematic, straightforward, nonapologetic, yet empathic manner. While it is necessary to implement the telling phase straightforwardly, the nurse's inability to keep their emotions in check is likely to impede the efficacy of the process. A perceived transfer of emotions from the nurse to the patient also takes place between the planning and response phases, which further requires increased competencies in emotional intelligence. Although balancing one’s stress levels and increasing anxiety on the part of the patient can be emotionally demanding for nurses, appropriate reflection-in-action during the response phase facilitates the development of self-awareness of the practitioners’ worldviews, which results in a successful follow-up for patients.

The use of protocols for breaking bad news has been explored as a model of good practice in health care. However, due to the fiduciary duty of nurses to patients, recommendations regarding breaking bad news require reflection on the emotional consequences of this task for nurses. Although emotional intelligence could be self-taught, the analysis of the literature on breaking bad news has shown that the development of a model that incorporates social and emotional learning into nursing practice is necessary to further enhance guidelines on breaking bad news. While nurses are likely to benefit from metacognitive strategies for delivering bad news, further studies focused on the development of interpersonal and social skills to aid healthcare professionals in dealing with the emotional commitment associated with communicating bad news are necessary to enhance professional development.
